# Physical education performance as a protective pathway: breaking the cycle between learning burnout and gaming disorder in Chinese adolescents

**DOI:** 10.3389/fpsyt.2026.1894271

**Published:** 2026-07-17

**Authors:** Qiurong Jiang, Cheng Wei, Man Chen, Xiaogang Wang, Guangrong Gong, Yaoyao Zhou, Qinghai Zou, Yisong Yang

**Affiliations:** 1 College of Education Chengdu College of Arts and Sciences, Chengdu, China; 2 School of Education and Psychology, Southwest Minzu Univers, Chengdu, China; 3 Dujiangyan Taziba Middle School, Dujiangyan, China

**Keywords:** academic performance, gaming disorder, learning burnout, middle school student, physical education performance

## Abstract

**Background and Aims:**

Gaming disorder (GD) and learning burnout (LB) are critical issues impacting adolescents, with GD recognized by the World Health Organization as a behavioral addiction and LB contributing to academic disengagement. While prior research has examined bivariate relationships, such as GD’s negative correlation with academic performance and physical education performance, or LB’s association with poor grades, no study has integrated all four variables into one model to explore their dynamic interactions longitudinally.

**Methods:**

A cross-lagged panel network (CLPN) model was applied to longitudinal data from 811 Chinese middle school students collected at two time points.

**Results:**

GD and LB showed minimal direct effects on academic performance or physical education performance. Instead, academic performance and physical education performance acted as protective factors, significantly alleviating LB, particularly among boys. These two protective factors were mutually reinforcing. Cognitive exhaustion (a core component of LB) and GD functioned as reciprocal risk factors.

**Conclusion:**

Interventions for adolescent maladjustment should prioritize addressing learning burnout through academic and physical avenues rather than overemphasizing gaming disorder, with tailored strategies for different genders.

## 1 Introduction

Gaming disorder (GD) has been added to the International Classification of Diseases, 11th Revision (ICD-11), by the World Health Organization (WHO) at 2020. It refers to excessive video gaming behavior characterized by impaired control over gaming, valuing it over other matters, and continued engagement in it despite knowledge of the adverse impact of it on physical, mental health and social functioning (1). A meta-analysis including 227,665 participants across 29 countries showed that the overall pooled prevalence of GD was 3.3% (2). Many studies have shown that age is a moderator of GD prevalence, adolescents with higher vulnerability (3, 4). The prevalence of GD among adolescents ranges from 4.6% (5) to 9.0% (6), and the rate is at a high level in East Asian (7). For example, a study among 503 adolescents (50.5% girls) in Hong Kong found that the prevalence was 15.1% (8), while among 1253 adolescents (43.8% girls) in Macao was 22.9% (9). What’s disturbing is that GD may cause functional impairment and distress among them (10), including poor mental health and physical health problems (11).

Recently, several studies have identified a negative correlation between GD and academic performance (12, 13). Because it is difficult to limit gaming time, a significant amount of time that should be spent on academic tasks, such as studying or doing homework, is instead devoted to gaming. In addition to GD, learning burnout (LB) is also an important factor contributing to lower academic performance (14). Based on the Conversation of Resource Theory (COR, 15), Zhu (2016) suggested that LB is a state of exhaustion that occurs among students during the learning process, including the feeling that emotional, cognitive, and physical energy is exhausted (15). Lacombe et al. (2023) summarized eight studies about LB among adolescents from 11 countries and found that the prevalence of LB ranged from 4.5% to 32.2% (16). And the 32.2% prevalence came from a study conducted in China, which obtained from 1209 middle school students (60.2% girls) in Hong Kong (17).

There is a Chinese proverb that says, “Everything is worthless except learning”, which means that learning takes an absolutely dominant role among Chinese students, and they face more severe academic competition and academic pressure compared to their peers in Western countries (18). The large amount of academic tasks may lead to a sense of fatigue and loss, and a decrease in self efficacy, which leads to a loss of interest in learning, that is LB (19). In such circumstances, students may look for ways to relieve stress and try to succeed in areas of interest to them. Games have a strong appeal to adolescents because of their immediate feedback and the opportunities for self-expression (7), could become an addictive outlet, potentially evolving into GD.

Worryingly, GD can not only undermine adolescents’ academic performance, but also their physical fitness (20). A meta-analysis found that GD may cause problems such as poor sleep, joint pain, headaches and vision loss, and these problems are more common in adolescents (21). Interestingly, enhancing sport and exercise is thought to be effective for alleviating GD (22). And a randomized controlled trial also demonstrated that aerobic training was effective in reducing the severity of GD compared to the control group (23). Another intervention with two groups of adolescents suffering from GD, group A using cognitive behavioral therapy (CBT) only, and group B performing CBT with physical activities, found that GD in group B decreased more significantly (24). In fact, physical activity not only helps to alleviate GD, but also reduces the negative effects of stress on health (25), and help to improve children’s executive and cognitive function (26), which in turn may improves students’ academic performance. For example, a study surveyed 259 children (60.2% girls) and found that aerobic exercise was positively associated with reading and math performance (27). In addition, lack of physical activity may be a risk factor for LB (17). Several studies have also indicated that physical activity can reduce LB (28, 29).

It is important to note that gender is one of the moderating factors of GD (30). Research has shown that boys and girls have different gaming preferences (31), with a meta-analysis (8) indicating a higher prevalence of GD among boys (6.8%) compared to girls (1.3%). This may be because boys have a stronger urge to play and spend more time playing compared to girls (32). In addition to GD, there are also gender differences in LB. Specifically, girls are more prone to burnout (33). Salmela-Aro and Tynkkynen (2012) suggested that girls have more academic stress and therefore tend to focus on poor performance and academic failure (34), which in turn increases burnout levels. As for the relationship between physical education performance and academic performance, boys and girls also differ. For example, the California Department of Education (2005) evaluated more than 1 million children and adolescents and found that the relationship between physical activity and academic performance was stronger for girls than for boys in the 7th grade (35).

The bivariate relationship between GD, LB, academic performance, and physical education performance has been thoroughly explored and consensus has been reached (12, 14). However, a notable gap exists, as studies integrating all four factors into one model have not yet emerged. Additionally, the majority of existing research on these aspects is cross-sectional and few are longitudinal, lacking the longitudinal depth necessary for elucidating their dynamic interactions. Fortunately, complex network theory provides both theoretical and methodological support (36). Within a network, variables are characterized as nodes, and the interactions between variables are characterized as the edges (37). To clarify the interactions between GD, LB, academic performance, and physical education performance, this study conducted two surveys among middle school students, and fitted the data with cross-lagged panel network model (CLPN). Recognizing the potential moderating effect of gender on these relationships, separate networks were constructed separately for boys and girls. CLPN is an approach of complex network theory in psychological research, It builds on data from two time points in a longitudinal design and construct unique cross-lagged relationships between multiple variables through a series of lasso regressions (38). With the help of network visualization methods, the study presents clear images depicting the cross-lagged relationships between variables, enhancing the readability, a methodology that has gained popularity in psychological research (39).

## 2 Methods

### 2.1 Participants and procedure

This study was conducted within a middle school in Shenzhen, Guangdong Province, China. Two surveys (6 months apart) were conducted on GD and LB, and both surveys were conducted with the help of Questionnaire Star, an online survey platform that can share QR codes, links or other methods of collection. The first survey (September 2022, T1) involved 947 students (mean age = 13.4 years, *SD* = 1.1; 590 boys, 357 girls; 320 seventh, 315 eighth, 312 ninth graders), of which 63 students spent no > 2 s on average to make their choices, and 18 students self reported not answering each item honestly and carefully. Thus, data from these 81 students were suggested to be excluded according to DeSimone and Harms (2018) (40). Of the remaining 866 students, 20 students did not participate or respond to the second survey, and only 846 of them (mean age = 13.5 years, *SD* = 1.1; 535 boys, 311 girls; grade distribution similar to T1) participated it (February 2023, T2), of which 35 students were also excluded for the same reason as T1. Therefore, the total number of students who participated in both surveys and had valid data was 811, including 511 boys, 294 seventh graders and 263 eighth graders. Both surveys were conducted with the consent of the local education bureau, as well as the school principal, while an informed consent form was signed by the students and their guardians before participating in the survey. Moreover, all materials and procedures were approved by the Research Ethics Committee of the corresponding author’s university.

### 2.2 Measures

#### 2.2.1 Academic performance and physical education performance

Students’ academic performance included three subjects: Chinese, English, and mathematics. These subjects are offered in both 7, 8 and 9 grade, and occupy the largest amount of school hours for Chinese middle school students. Students’ physical education performance was assessed through their physical education exam scores, which are tested every semester for middle school students, with 1000 meter runs and pull−ups for boys, 800 meter runs and sit−ups for girls. Although these scores directly measure physical fitness rather than habitual physical activity, they serve as a reasonable proxy for physical activity engagement in the Chinese educational context. The high school entrance examination mandates PE scores as a compulsory component, and students cannot achieve satisfactory results without sustained daily training and active participation in physical education classes throughout the school year. Thus, while we did not capture step counts or leisure−time exercise, our PE performance indicator reflects the cumulative behavioral engagement that the educational system explicitly incentivizes. Since students were tested for academic and physical education performance only at the end of each semester, the first academic and physical education performance test in this study was in July 2022, and the second was in January 2023. For the purpose of analysis, the academic and physical education performance of the first time was treated as the first wave of data along with LB and GD at T1, and the second time was treated as the second wave of data along with LB and GD at T2.

#### 2.2.2 9-item internet gaming disorder scale (IGDS9-SF)

The Nine-Item Internet Gaming Disorder Scale (IGDS9-SF) (41) was used in this study to measure gaming disorder among middle school students. This scale has 9 items on a 5-point scale from 1 (never) to 5 (always) and has good reliability and validity among Chinese adolescents (42). The Cronbach’s alpha for IGDS9-SF was 0.90 (boys) and 0.92 (girls) at T1 and 0.91 (both boys and girls) at T2. A score of 32 or above (43) on the IGDS9-SF is considered indicative of gaming disorder.

#### 2.2.3 Learning burnout questionnaire for middle school students

This study used the Learning Burnout Questionnaire for Middle School Students developed by (15) based on the COR (44) to measure learning burnout. This questionnaire contains 3 factors, emotional exhaustion, cognitive exhaustion and physical exhaustion, with a total of 20 questions, using a 5-point scale from 0 (not at all) to 4 (fully). At T1, The Cronbach’s alpha for emotional exhaustion was 0.91 (boys) and 0.92 (girls), cognitive exhaustion was 0.92 (boys) and 0.93 (girls), physical exhaustion was 0.84 (both boys and girls). At T2, The Cronbach’s alpha for emotional exhaustion was 0.93 (both boys and girls), cognitive exhaustion was 0.93 (boys) and 0.94 (girls), physical exhaustion was 0.85 (boys) and 0.84 (girls).

### 2.3 Data analysis

#### 2.3.1 Standardized student academic and physical education performance

The test scores of Chinese, English, and mathematics were standardized within each grade, and the standardized scores of the 3 subjects were summed to obtain the students’ academic performance. Similar to academic performance, students’ physical education performance was standardized by gender within grade levels.

#### 2.3.2 Network estimation and visualization

The CLPN consists of a series of regression equations that calculate the autoregressive (a variable at T1 predicting itself at T2 after controlling for all other variables at T1) and cross-lagged relationships (a variable at T1 predicting a different variable at T2 after controlling for all other variables at T1) for each variable in the network. To minimize the false positive edges, this study also adopted a 10-fold cross validation tuning parameter selection method to regularize the regression coefficients with the help of LASSO. CLPN can be visualized, and every variable corresponds to a node. Thicker and more saturated edges represent stronger connections, and the blue solid edges epresent positive regression coefficients, while the red dashed edges represent the opposite. For both networks, the minimum edge value is set to 0, and the maximum edge value is set at 0.40 (absolute value), which is the maximum edge of two networks (excluding autoregressive edges).

#### 2.3.3 Accuracy estimation

An bootstrap method in the R package *bootnet* (45) were used to test the accuracy of the network. With the help of a nonparametric bootstrap of 1000 instances, this study tested its accuracy by calculating 95% confidence intervals (CIs) for each edge weight. The wider the 95% CIs for the edge weights, the more caution should be taken in interpreting. Subsequently testing whether the weights between edges are significantly non-zero (*α* = 0.05).

#### 2.3.4 Network comparison

In this study, Pearson’s correlations between the boys’ and girls’ network were first calculated, while Mann-Whitney *U* tests were later performed on the weights of edges with the help of bootstrapping (46). Vargha and Delaney’s A was used to determine the effect sizes for the nonparametric tests, with 056–0.64 or 0.34–0.44 being small effect sizes; 0.64–0.71 or 0.29–0.34 being moderate effect sizes; and ≥0.71 or ≤0.29 being large effect sizes (47).

## 3 Results

In the initial sample of 947 students, 81 were excluded for invalid responses. Of the remaining 866, 20 did not participate at T2, and 35 were further excluded for poor quality, yielding a final analytical sample of 811 completers. For the 55 non−completers, listwise deletion was applied, as required by the bootnet package. Baseline comparisons showed no significant differences between completers and non−completers on all study variables (*p* > 0.05), indicating random attrition and minimising bias.

**Figure 1:**
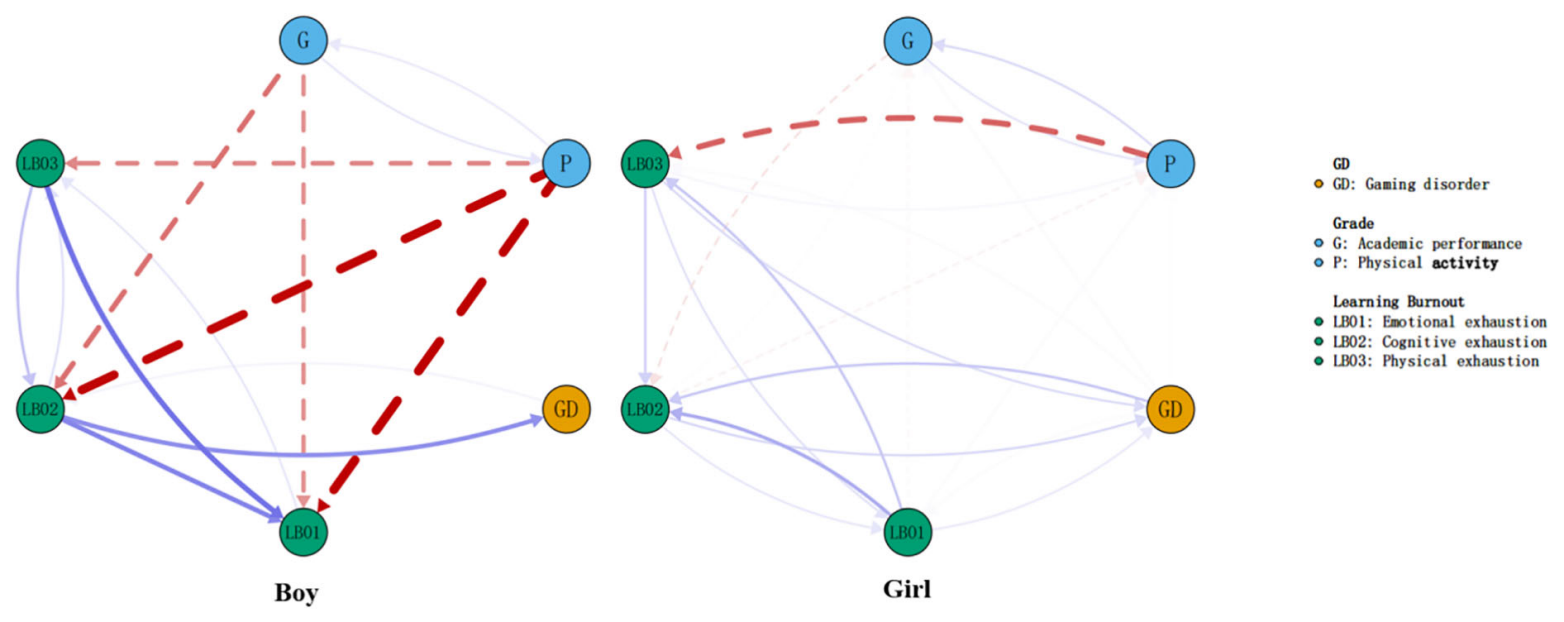
CLPN for boys and girls.

**Table 1** presents the descriptive statistics between the variables. **Figure 1** shows the network structure of boys and girls, and the weights of each edge are shown in **Table 2**. It can be seen that the connection within boys is stronger, and the autoregressive edges are not presented in **Figure 1** because they are significantly larger than the cross-lagged edges. The findings indicate that GD and LB had little direct impact on students’ academic performance. Among boys, academic performance had a strong alleviating effect on emotional and cognitive exhaustion, but this effect was not pronounced among girls. In addition, academic performance and physical education performance are mutually beneficial, both are contributing factors to the other. Students’ physical education performance has a strong alleviating effect on LB, and there are gender differences. The relationship between physical education performance and GD is weak. LB and GD both are risk factors for each other. The 95% confidence intervals (CIs) for all edge weights are presented in the online (**Supplementary Figure S1**). Although the 95% CIs for most edges showed substantial overlap, the weights of the stronger edges were clearly distinguishable from those of the weaker edges. The results of pairwise comparisons testing for differences between specific edge weights are detailed in the (**Supplementary Figure S2**), revealing some meaningful distinctions.

**Table 1:** The descriptive statistics.

Variable	*M*				*SD*				Skewness				Kurtosis			
	Wave1		Wave2		Wave1		Wave2		Wave1		Wave2		Wave1		Wave2	
	Boy	Girl	Boy	Girl	Boy	Girl	Boy	Girl	Boy	Girl	Boy	Girl	Boy	Girl	Boy	Girl
Gaming disorder	16.43	15.45	16.09	15.19	7.28	7.42	7.19	6.76	1.06	1.46	1.30	1.17	0.91	2.02	1.86	0.98
Learning burnout	12.86	16.85	13.30	17.84	14.17	17.34	15.31	17.50	1.33	1.33	1.63	1.08	1.36	1.38	3.03	0.57
Emotional exhaustion	5.20	6.67	5.17	7.07	6.07	7.51	6.48	7.48	1.25	1.34	1.66	1.10	0.89	1.26	2.84	0.51
Cognitive exhaustion	5.13	6.74	5.37	6.72	6.12	7.01	6.34	7.03	1.32	1.12	1.45	1.06	1.10	0.59	1.75	0.34
Physical exhaustion	2.52	3.44	2.76	4.04	3.35	4.11	3.63	4.27	1.90	1.55	1.95	1.25	4.44	2.26	4.78	1.30
Chinese	81.41	86.42	80.81	85.75	12.90	9.60	12.31	10.24	-1.32	-1.60	-1.29	-1.47	2.91	3.46	3.94	4.99
English	77.57	75.49	73.12	69.82	18.22	17.98	18.39	17.95	-1.41	-1.22	-1.21	-0.98	1.50	1.15	1.24	0.43
Mathematics	69.20	75.07	64.89	70.06	21.20	17.08	21.13	17.45	-0.73	-0.94	-0.50	-0.74	-0.44	0.49	-0.80	-0.08
Physical education	30.24	38.36	39.74	41.47	12.43	9.96	6.49	5.76	-0.15	-1.09	-1.58	-1.90	-0.92	0.99	4.91	4.24

**Table 2:** The weights of each edge in tow networks.

Boy							Girl						
Node	G	P	GD	LB01	LB02	LB03		G	P	GD	LB01	LB02	LB03
G	0.972	0.039	0.000	-0.165	-0.216	0.000	G	0.975	0.040	0.000	0.000	-0.044	0.000
P	0.034	0.336	0.000	-0.397	-0.388	-0.185	P	0.055	0.377	0.000	0.000	0.000	-0.242
GD	0.000	0.000	0.373	0.000	0.023	0.000	GD	0.008	0.002	0.444	0.006	0.080	0.008
LB01	0.000	0.000	0.000	0.287	0.000	0.038	LB01	-0.015	0.007	0.035	0.447	0.122	0.081
LB02	0.000	0.000	0.169	0.186	0.468	0.049	LB02	-0.005	-0.025	0.058	0.037	0.309	0.000
LB03	0.000	0.000	0.000	0.216	0.092	0.343	LB03	0.000	0.016	0.053	0.044	0.069	0.362

The Pearson’s correlation between the networks for boys and girls was 0.87, and the results of the Mann-Whitney U test for the weights of each edge in the network are shown in **Table 3**. It can be found that the results obtained after 1000 bootstrapping on the sample are consistent with **Table 1**, most of the differences between boys and girls are real, with above moderate effect sizes.

**Table 3:** The Mann-Whitney *U* test for the weights of each edge.

Edge	Boy	Girl	Z	*p*	Effect sizes
G → P	0.041(0.029~0.050) ^a^	0.026(0.000~0.045)	11.472	0.000	0.200
G → GD	0.000(-0.091~0.000)	0.000(0.000~0.000)	–	–	–
G → LB01	-0.160(-0.219~0.090)	0.000(0.000~0.000)	30.096	0.000	0.134
G → LB02	-0.216(-0.275~0.146)	0.000(-0.124~0.000)	23.606	0.000	0.201
G → LB03	0.000(0.000~0.000)	0.000(0.000~0.000)	–	–	–
P → G	0.023(0.000~0.054)	0.000(0.000~0.070)	2.443	0.015	0.530
P → GD	0.000(-0.152~0.000)	0.000(0.000~0.000)	–	–	–
P → LB01	-0.401(-0.521~0.256)	0.000(0.000~0.000)	31.811	0.000	0.116
P → LB02	-0.380(-0.488~0.219)	0.000(0.000~0.000)	34.409	0.000	0.079
P → LB03	-0.153(-0.218~0.000)	-0.155(-0.322~0.000)	1.882	0.060	–
GD → G	0.000(0.000~0.000)	0.000(0.000~0.007)	–	–	–
GD → P	0.000(0.000~0.000)	0.000(0.000~0.006)	–	–	–
GD → LB01	0.000(0.000~0.000)	0.000(0.000~0.033)	–	–	–
GD → LB02	0.020(0.000~0.047)	0.074(0.036~0.103)	19.985	0.000	0.243
GD → LB03	0.000(0.000~0.000)	0.000(0.000~0.018)	–	–	–
LB01 → G	0.000(0.000~0.000)	0.000(-0.012~0.000)	–	–	–
LB01 → P	0.000(0.000~0.000)	0.000(0.000~0.001)	–	–	–
LB01 → GD	0.000(0.000~0.000)	0.029(0.000~0.068)	28.293	0.000	0.172
LB01 → LB02	0.000(0.000~0.000)	0.119(0.065~0.163)	35.202	0.000	0.064
LB01 → LB03	0.033(0.000~0.058)	0.069(0.038~0.092)	16.411	0.000	0.289
LB02 → G	0.000(0.000~0.000)	0.000(-0.006~0.000)	–	–	–
LB02 → P	0.000(-0.003~0.000)	0.000(-0.022~-0.003)	–	–	–
LB02 → GD	0.173(0.138~0.220)	0.049(0.000~0.082)	30.776	0.000	0.897
LB02 → LB01	0.193(0.153~0.231)	0.024(0.000~0.067)	32.834	0.000	0.923
LB02 → LB03	0.044(0.021~0.067)	0.000(0.000~0.010)	26.150	0.000	0.828
LB03 → G	0.000(0.000~0.000)	0.000(0.000~0.000)	–	–	–
LB03 → P	0.000(0.000~0.000)	0.000(0.000~0.016)	–	–	–
LB03 → GD	0.000(0.000~0.026)	0.040(0.000~0.094)	13.303	0.000	0.340
LB03 → LB01	0.214(0.148~0.285)	0.033(0.000~0.121)	23.889	0.000	0.307
LB03 → LB02	0.101(0.012~0.173)	0.044(0.000~0.131)	7.486	0.000	0.596

^a^ 0.041(0.029~0.050), 0.041 is the median of 1000 bootstrap samples, 0.029 is the 25th percentile and 0.050 is the 75th percentile.

## 4 Discussion

This study is the first to model GD, LB, academic performance, and physical function in one model and quantifies the interactions between them with the help of CLPN. It was found that GD had no effect on students’ academic performance and physical education performance. Students’ academic performance and physical education performance effectively relieved their LB, especially among boys. Academic performance and physical education performance are mutually beneficial, while the cognitive exhaustion and GD are risk factors for each other.

Results of this study suggest that the widespread belief among Chinese parents that video games are great scourges may be biased. Several studies have also confirmed that playing video games has little or no effect on students’ academic performance (13, 48). A survey conducted with 1,502 adolescents (49.9% girls) showed that students who played video games occasionally and moderately had better academic performance, while the opposite was true for those who played video games frequently (49). Parents often ignore children’s proper reasons for playing video games (50), focusing only on the time they spend and other activities they give up because of it. In fact, even when kids aren’t playing video games, that time is spent on TV, smart phones, comic books, and unstructured sports. In addition, there may be positive effects of playing video games, which require cognitive skills such as concentration and memory. Studies have shown that hand-eye coordination, math skills, and language skills are improved during play video games (51). However, this null finding must be interpreted with considerable caution. Our community sample was largely subclinical, with most students unlikely to meet the clinical threshold for gaming disorder according to the IGDS9−SF cutoff, and the restricted range of symptom severity may have attenuated any potential association with test scores. Moreover, standardized exam scores capture point−in−time competency and may be less sensitive to the cumulative erosion of study habits or homework quality than teacher−assigned grades or longitudinal GPA. The Chinese government’s mandatory anti−addiction system for minors also limits daily playtime on popular online platforms, which may have weakened the GD−performance link observed in other contexts. We therefore frame this finding not as evidence that GD is academically harmless, but as a tentative observation challenging prevailing assumptions that requires replication in clinical samples and with more ecologically valid academic measures.

Another finding of this study was that there was no relationship between GD and physical education performance. However, most studies have concluded that GD impairs physical health and that improving physical education performance can alleviate GD (22, 24, 52). One possible explanation is that, many online video games popular among Chinese students have been mandated by the Chinese government to include anti-addiction mechanisms for minors, which objectively reduces the amount of time students spend playing games and thus mitigates the damage that GD on physical education performance. Another thing to note is that at the time of T1, China had not yet removed the pandemic warning for the COVID-19, and even students in Shenzhen had to study at home due to the pandemic, and students’ physical education classes and outdoor exercise were restricted. So, lack of adequate physical education performance leads to ineffective GD relief.

This study also found that LB had a negligible effect on students’ academic performance, but that academic performance had a mitigating effect on LB. It is a common knowledge that LB can have a negative effect on a student’s academic performance. For example, a meta-analysis included 29 studies and more than 10,000 adolescents, found a negative relationship between LB and academic performance (14). However, one study with similar results to ours found that LB has no direct effect on academic performance (53). They explained this by suggesting that other factors may mediate the effect of LB on academic performance, such as disturbed sleep. Another possible reason is the study design, previous studies have been mostly cross-sectional (54, 55), which may have yielded a negative relationship between LB and academic performance, but the direction of this relationship cannot be determined.

Does LB make students’ academic performance worse, or does students’ academic performance help alleviate LB? With the help of the CLPN, this study found that academic performance was a precursor to LB and that better grades were associated with less LB, which was particularly pronounced among boys. The “gain and loss spirals” of demand-resource theory suggests that higher performing students tend to invest more energy and resources in school and are more motivated to stay highly engaged in school, thus entering a “gain spiral” (56). In contrast, lower performing students may enter a “loss spiral” (57). As for gender differences, Eccles (2007) suggested that boys are more motivated by the pursuit of success and competition, while girls are more motivated by help-seeking and social support (58). Elliot and Church (1997) also found that boys have higher motivation in pursuit of success and a more significant negative correlation with LB than girls (59). Thus, for boys, improved academic performance may enhance their motivation to learn and thus reduce LB, whereas for girls, access to social support and help may have a more ameliorative effect on LB.

Consistent with other studies (28, 60), this study also found that physical education performance was a protective factor for academic performance. Cross-stressor-adaptation (CSA) hypothesis argues that physical education performance enhances human adaptation, including but not limited to cognitive processes, social adaptation, and psychological well-being (61). Compared to boys, girls have poorer skeletal, muscular and cardiorespiratory fitness (62), while girls have higher levels of academic stress and LB (33, 63). This may be one of the reasons for the little effect of physical education performance on LB in girls’ network. Cognitive exhaustion (one factor of LB) and GD are each other’s risk factors for both boys and girls. Students with high levels of cognitive exhaustion usually need to engage in relaxation and self-regulation activities to relieve academic stress. However, if they play games, they are likely to develop a dependence on games and withdrawal difficulties, which eventually lead to GD (64).

Despite the novelty and valuable findings of this study, several limitations warrant careful consideration. First, the single school and single city design in Shenzhen, a highly affluent and technologically saturated urban hub, severely restricts generalizability to rural, lower income, or less digitally immersed regions of China and other countries. The intense academic competition and extensive digital infrastructure unique to Shenzhen may have amplified or moderated the observed relationships, limiting external validity. Second, we acknowledge a conceptual mismatch in our physical activity measure; physical education exam scores, comprising specific fitness tests, directly assess cardiorespiratory endurance and muscular strength rather than habitual daily energy expenditure, step counts, or leisure time exercise. While these scores serve as a reasonable proxy within the Chinese educational context where sustained training is required for high exam performance, this operationalization may attenuate or alter associations compared to behavioral measures. Third, a critical temporal misalignment existed between the collection of academic and physical education scores (end of semester) and questionnaire data (beginning of semester), resulting in cross lagged pathways spanning unequal intervals (e.g., 7 months vs. 4 months). This asymmetry may bias direct comparisons of edge weights, potentially overemphasizing academic performance as a predictor of learning burnout relative to reverse pathways. Fourth, our community sample was predominantly subclinical regarding gaming disorder, and the Chinese government’s mandatory anti addiction system for minors may have artificially reduced gaming time, both of which could dilute the observed effect of GD on academic outcomes. Consequently, our null findings should not be interpreted as conclusive evidence that GD is academically harmless but rather as tentative observations requiring replication in clinical populations. Fifth, the assessment of gaming disorder and learning burnout relied entirely on self report, introducing social desirability bias, while standardized test scores, though objective, may be less sensitive to cumulative declines in study habits compared to teacher assigned grades or longitudinal GPA. We confirmed random attrition via baseline comparisons, yet the application of listwise deletion for missing data remains a methodological constraint. Finally, the longitudinal design, although a strength, was confined to only two time points over six months, precluding fine grained examination of within person fluctuations and feedback loops. Future research should employ intensive longitudinal designs such as ecological momentary assessment, incorporate objective physical activity trackers, include diverse multiregional samples, and align measurement intervals to validate and extend our network findings.

## Data Availability

The raw data supporting the conclusions of this article will be made available by the authors, without undue reservation.

## References

[1] World Health Organization . 6C51 Gaming Disorder (2020). Available online at: https://icd.who.int/browse11/l-m/en#/http://id.who.int/icd/entity/1448597234 (Accessed June 30, 2026).

[2] Kim HS Son G Roh EB Ahn WY Kim J Shin SH . Prevalence of gaming disorder: A meta-analysis. Addict Behav. (2022) 126:107183. doi: 10.1016/j.addbeh.2021.107183 34864436

[3] Mentzoni RA Brunborg GS Molde H Myrseth H Skouveroe KJM Hetland J . Problematic video game use: Estimated prevalence and associations with mental and physical health. Cyberpsychol Behav Soc Netw. (2011) 14:591–6. doi: 10.1089/cyber.2010.0260 21342010

[4] Vollmer C Randler C Horzum MB Ayas T . Computer game addiction in adolescents and its relationship to chronotype and personality. SAGE Open. (2014) 4(1):2158244013518054. doi: 10.1177/2158244013518054

[5] Fam JY . Prevalence of internet gaming disorder in adolescents: A meta-analysis across three decades. Scand J Psychol. (2018) 59:524–31. doi: 10.1111/sjop.12459 30004118

[6] Gentile DA Bailey K Bavelier D Brockmyer JF Cash H Coyne SM . Internet gaming disorder in children and adolescents. Pediatrics. (2017) 140:S81–5. doi: 10.1542/peds.2016-1758H 29093038

[7] Paulus FW Ohmann S von Gontard A Popow C . Internet gaming disorder in children and adolescents: a systematic review. Dev Med Child Neurol. (2018) 60:645–59. doi: 10.1111/dmcn.13754 29633243

[8] Wang CW Chan CLW Mak KK Ho SY Wong PWC Ho RTH . Prevalence and correlates of video and internet gaming addiction among Hong Kong adolescents: a pilot study. Sci World J. (2014), 874648. doi: 10.1155/2014/874648 25032242 PMC4083269

[9] Chen JH Su XY Dang L Wu AMS . Evaluation of the psychometric properties of the Chinese internet gaming disorder checklist (C-IGDC) among Chinese adolescents. Front Psychiatry. (2021) 12:721397. doi: 10.3389/fpsyt.2021.721397 34589007 PMC8473869

[10] Muller KW Janikian M Dreier M Wolfling K Beutel ME Tzavara C . Regular gaming behavior and internet gaming disorder in European adolescents: results from a cross-national representative survey of prevalence, predictors, and psychopathological correlates. Eur Child Adolesc Psychiatry. (2015) 24:565–74. doi: 10.1007/s00787-014-0611-2 25189795

[11] Mannikko N Billieux J Kaariainen M . Problematic digital gaming behavior and its relation to the psychological, social and physical health of Finnish adolescents and young adults. J Behav Addict. (2015) 4:281–8. doi: 10.1556/2006.4.2015.040 26690623 PMC4712762

[12] Adelantado-Renau M Moliner-Urdiales D Cavero-Redondo I Beltran-Valls MR Martinez-Vizcaino V Alvarez-Bueno C . Association between screen media use and academic performance among children and adolescents: a systematic review and meta-analysis. JAMA Pediatr. (2019) 173:1058–67. doi: 10.1001/jamapediatrics.2019.3176 31545344 PMC6764013

[13] Ferguson CJ . Do angry birds make for angry children? A meta-analysis of video game influences on children's and adolescents' aggression, mental health, prosocial behavior, and academic performance. Perspect Psychol Sci. (2015) 10:646–66. doi: 10.1177/1745691615592234 26386002

[14] Madigan DJ Curran T . Does burnout affect academic achievement? A meta-analysis of over 100,000 students. Educ Psychol Rev. (2021) 33:387–405. doi: 10.1007/s10648-020-09533-1 30311153

[15] Zhu Z . The development and application of high school students' learning burnout questionnaire. Beijing Normal University, Beijing (2016).

[16] Lacombe N Hey M Hofmann V Pagnotta C Squillaci M . School burnout after COVID-19, prevalence and role of different risk and protective factors in preteen students. Children-Basel. (2023) 10:823. doi: 10.3390/children10050823 37238371 PMC10217600

[17] Cheung P Li CX . Physical activity and mental toughness as antecedents of academic burnout among school students: a latent profile approach. Int J Environ Res Public Health. (2019) 16:2024. doi: 10.3390/ijerph16112024 31174377 PMC6603857

[18] Cheng XC Lin HL . Mechanisms from academic stress to subjective well-being of Chinese adolescents: the roles of academic burnout and internet addiction. Psychol Res Behav Manag. (2023) 16:4183–96. doi: 10.2147/prbm.S423336 37868651 PMC10590069

[19] Salmela-Aro K Kiuru N Leskinen E Nurmi JE . School Burnout Inventory (SBI) reliability and validity. Eur J Psychol Assess. (2009) 25:48–57. doi: 10.1027/1015-5759.25.1.48

[20] Byeon G Jo SJ Park JI Jeong H Lee HK Yim HW . Risk factors and outcomes of internet gaming disorder identified in Korean prospective adolescent cohort study. J Behav Addict. (2022) 11:1035–43. doi: 10.1556/2006.2022.00071 36194504 PMC9881665

[21] Benchebra L Alexandre JM Dubernet J Fatseas M Auriacombe M . Gambling and gaming disorders and physical health of players: a critical review of the literature. Presse Med. (2019) 48:1551–68. doi: 10.1016/j.lpm.2019.10.014 31767247

[22] Henchoz Y Studer J Deline S N'Goran AA Baggio S Gmel G . Video gaming disorder and sport and exercise in emerging adulthood: a longitudinal study. Behav Med. (2016) 42:105–11. doi: 10.1080/08964289.2014.965127 25258243

[23] Maden C Bayramlar K Aricak OT Yagli NV . Effects of virtual reality-based training and aerobic training on gaming disorder, physical activity, physical fitness, and anxiety: a randomized, controlled trial. Ment Health Phys Act. (2022) 23:100465. doi: 10.1016/j.mhpa.2022.100465 38826717

[24] Hong JS Kim SM Kang KD Han DH Kim JS Hwang H . Effect of physical exercise intervention on mood and frontal alpha asymmetry in internet gaming disorder. Ment Health Phys Act. (2020) 18:100318. doi: 10.1016/j.mhpa.2020.100318 38826717

[25] Wood CJ Clow A Hucklebridge F Law R Smyth N . Physical fitness and prior physical activity are both associated with less cortisol secretion during psychosocial stress. Anxiety Stress Coping. (2018) 31:135–45. doi: 10.1080/10615806.2017.1390083 29037088

[26] Li JW O'Connor H O'Dwyer N Orr R . The effect of acute and chronic exercise on cognitive function and academic performance in adolescents: A systematic review. J Sci Med Sport. (2017) 20:841–8. doi: 10.1016/j.jsams.2016.11.025 28185806

[27] Castelli DM Hillman CH Buck SM Erwin HE . Physical fitness and academic achievement in third and fifth grade students. J Sport Exerc Psychol. (2007) 29:239–52. doi: 10.1123/jsep.29.2.239 17568069

[28] Chen K Liu FY Mou L Zhao PT Guo LY . How physical exercise impacts academic burnout in college students: the mediating effects of self-efficacy and resilience. Front Psychol. (2022) 13:964169. doi: 10.3389/fpsyg.2022.964169 36438387 PMC9691659

[29] Souza R Guilherme RF Elias RGM Dos Reis LL Garbin de Souza OA Robert Ferrer M . Associated determinants between evidence of burnout, physical activity, and health behaviors of university students. Front Sports Act Living. (2021) 3:733309. doi: 10.3389/fspor.2021.733309 34746775 PMC8568456

[30] Marraudino M Bonaldo B Vitiello B Bergui GC Panzica G . Sexual differences in internet gaming disorder (IGD): from psychological features to neuroanatomical networks. J Clin Med. (2022) 11:1018. doi: 10.3390/jcm11041018 35207293 PMC8877403

[31] Homer BD Hayward EO Frye J Plass JL . Gender and player characteristics in video game play of preadolescents. Comput Hum Behav. (2012) 28:1782–9. doi: 10.1016/j.chb.2012.04.018 38826717

[32] Desai RA Krishnan-Sarin S Cavallo D Potenza MN . Video-gaming among high school students: health correlates, gender differences, and problematic gaming. Pediatrics. (2010) 126:E1414–24. doi: 10.1542/peds.2009-2706 21078729 PMC3678538

[33] Vansoeterstede A Cappe E Lichtle J Boujut E . A systematic review of longitudinal changes in school burnout among adolescents: trajectories, predictors, and outcomes. J Adolesc. (2023) 95:224–47. doi: 10.1002/jad.12121 36385709

[34] Salmela-Aro K Tynkkynen L . Gendered pathways in school burnout among adolescents. J Adolesc. (2012) 35:929–39. doi: 10.1016/j.adolescence.2012.01.001 22300678

[35] California Department of Education . A Study of the Relationship Between Physical Fitness and Academic Achievement in California Using 2004 Test Results. Sacramento, CA: California Department of Education (2005).

[36] Borsboom D . A network theory of mental disorders. World Psychiatry. (2017) 16:5–13. doi: 10.1002/wps.20375 28127906 PMC5269502

[37] Costantini G Epskamp S Borsboom D Perugini M Mõttus R Waldorp LJ . State of the aRt personality research: a tutorial on network analysis of personality data in R. J Res Pers. (2015) 54:13–29. doi: 10.1016/j.jrp.2014.07.003 38826717

[38] Rhemtulla M Cramer AOJ van Bork R Williams DR . Cross-lagged network models (2019). Available online at: https://osf.io/9h5nj/ (Accessed June 30, 2026).

[39] Xu B Yuan H Wu X Wang W . Comorbidity patterns of posttraumatic stress disorder and depression symptoms: cross-validation in two postearthquake child and adolescent samples. Depress Anxiety. (2023) 2023:4453663. doi: 10.1155/2023/4453663 40224591 PMC11921845

[40] DeSimone JA Harms PD . Dirty Data: The effects of screening respondents who provide low-quality data in survey research. J Bus Psychol. (2018) 33:559–77. doi: 10.1007/s10869-017-9514-9 30311153

[41] Pontes HM Griffiths MD . Measuring DSM-5 internet gaming disorder: development and validation of a short psychometric scale. Comput Hum Behav. (2015) 45:137–43. doi: 10.1016/j.chb.2014.12.006 38826717

[42] Yam CW Pakpour AH Griffiths MD Yau WY Lo CLM Ng JMT . Psychometric testing of three chinese online-related addictive behavior instruments among Hong Kong university students. Psychiatr Q. (2019) 90:117–28. doi: 10.1007/s11126-018-9610-7 30328020

[43] Qin LX Cheng LM Hu MR Liu QS Tong JQ Hao W . Clarification of the cut-off score for nine-item internet gaming disorder scale-short form (IGDS9-SF) in a Chinese context. Front Psychiatry. (2020) 11:470. doi: 10.3389/fpsyt.2020.00470 32528331 PMC7262730

[44] Hobfoll SE Shirom A . Conservation of resources theory: applications to stress and management in the workplace. In: Golembiewski RT , editor. Handbook of Organization Behavior. Marcel Dekker, New York (2000). p. 57–81.

[45] Epskamp S Borsboom D Fried EI . Estimating psychological networks and their accuracy: a tutorial paper. Behav Res Methods. (2018) 50:195–212. doi: 10.3758/s13428-017-0862-1 28342071 PMC5809547

[46] Liu YM Yuan H Song C Li LY Zhou WY Wang WC . Symptom relationships between internet addiction and anxiety across primary and middle school students during the Omicron lockdown. J Affect Disord. (2023) 329:251–6. doi: 10.1016/j.jad.2023.02.074 36828145

[47] Vargha A Delaney HD . A critique and improvement of the CL common language effect size statistics of McGraw and Wong. J Educ Behav Stat. (2000) 25:101–32. doi: 10.3102/10769986025002101 38293548

[48] Drummond A Sauer JD . Video games do not negatively impact adolescent academic performance in science, mathematics or reading. PLoS One. (2014) 9:e87943. doi: 10.1371/journal.pone.0087943 24699536 PMC3974676

[49] Gomez-Gonzalvo F Devis-Devis J Molina-Alventosa P . Video game usage time in adolescents' academic performance. Comunicar. (2020) 28:87–96. doi: 10.3916/c65-2020-08

[50] Kardefelt-Winther D . A critical account of DSM-5 criteria for internet gaming disorder. Addict Res Theory. (2015) 23:93–8. doi: 10.3109/16066359.2014.935350

[51] Barlett CP Vowels CL Shanteau J Crow J Miller T . The effect of violent and non-violent computer games on cognitive performance. Comput Hum Behav. (2009) 25:96–102. doi: 10.1016/j.chb.2008.07.008 38826717

[52] Moore S Satel J Pontes HM . Investigating the role of health factors and psychological well-being in gaming disorder. Cyberpsychol Behav Soc Netw. (2022) 25:94–100. doi: 10.1089/cyber.2021.0050 34788152

[53] Evers K Chen SF Rothmann S Dhir A Pallesen S . Investigating the relation among disturbed sleep due to social media use, school burnout, and academic performance. J Adolesc. (2020) 84:156–64. doi: 10.1016/j.adolescence.2020.08.011 32920331

[54] Cadime I Pinto AM Lima S Rego S Pereira J Ribeiro I . Well-being and academic achievement in secondary school pupils: The unique effects of burnout and engagement. J Adolesc. (2016) 53:169–79. doi: 10.1016/j.adolescence.2016.10.003 27814494

[55] Supervia PU Bordas CS . Burnout, goal orientation and academic performance in adolescent students. Int J Environ Res Public Health. (2020) 17:6507. doi: 10.3390/ijerph17186507 32906738 PMC7559595

[56] Hobfoll SE Halbesleben J Neveu JP Westman M . Conservation of resources in the organizational context: the reality of resources and their consequences. Annu Rev Organ Psychol Organ Behav. (2018) 5:103–28. doi: 10.1146/annurev-orgpsych-032117-104640 41139587

[57] Fiorilli C De Stasio S Di Chiacchio C Pepe A Salmela-Aro K . School burnout, depressive symptoms and engagement: their combined effect on student achievement. Int J Educ Res. (2017) 84:1–12. doi: 10.1016/j.ijer.2017.04.001 38826717

[58] Eccles JS . Where are all the women? Gender differences in participation in physical science and engineering. Washington, DC: American Psychological Association (2007). doi: 10.1037/11546-016

[59] Elliot AJ Church MA . A hierarchical model of approach and avoidance achievement motivation. J Pers Soc Psychol. (1997) 72:218–32. doi: 10.1037/0022-3514.72.1.218 10234849

[60] Tong JJ Zhang ZJ Chen WQ He ZH Yang XJ . How physical fitness influences academic burnout in elementary students: an interpersonal perspective. Curr Psychol. (2021) 42:5977–85. doi: 10.1007/s12144-021-01948-5 30311153

[61] Kjaer M . Regulation of hormonal and metabolic responses during exercise in humans. Exerc Sport Sci Rev. (1992) 20:161–84. doi: 10.1249/00003677-199200200-00006 1623885

[62] Boraita RJ Ibort G Torres JMD Alsina DA . Gender differences relating to lifestyle habits and health-related quality of life of adolescents. Child Indic Res. (2020) 13:1937–51. doi: 10.1007/s12187-020-09728-6 30311153

[63] Vinter K Aus K Arro G . Adolescent girls' and boys' academic burnout and its associations with cognitive emotion regulation strategies. Educ Psychol. (2021) 41:1061–77. doi: 10.1080/01443410.2020.1855631 37339054

[64] Andreassen CS Billieux J Griffiths MD Kuss DJ Demetrovics Z Mazzoni E . The relationship between addictive use of social media and video games and symptoms of psychiatric disorders: a large-scale cross-sectional study. Psychol Addict Behav. (2016) 30:252–62. doi: 10.1037/adb0000160 26999354

