# Supplementary material for: Physical education performance as a protective pathway: breaking the cycle between learning burnout and gaming disorder in Chinese adolescents
Source: Front Psychiatry. 2026 Jul 17;17:1894271. doi: 10.3389/fpsyt.2026.1894271 (PMC13423978; doi:10.3389/fpsyt.2026.1894271)
Supplement: Supplementary file 1 [file SupplementaryFile1.docx]

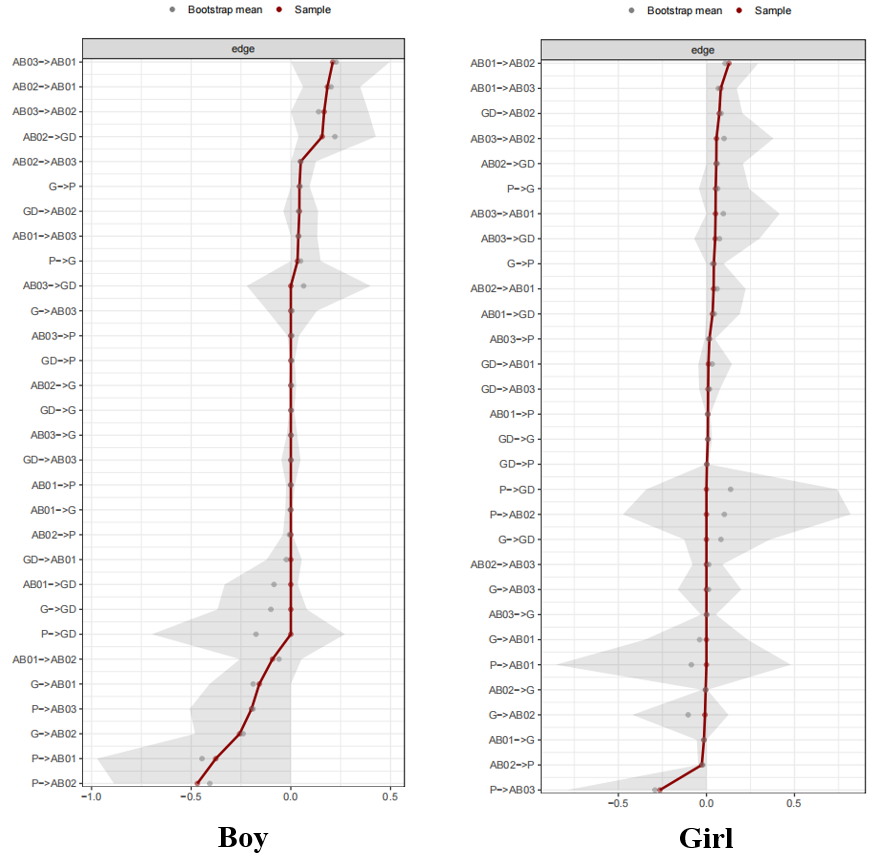


Fig. S1 The bootstrapped 95 % CIs for all edge weights


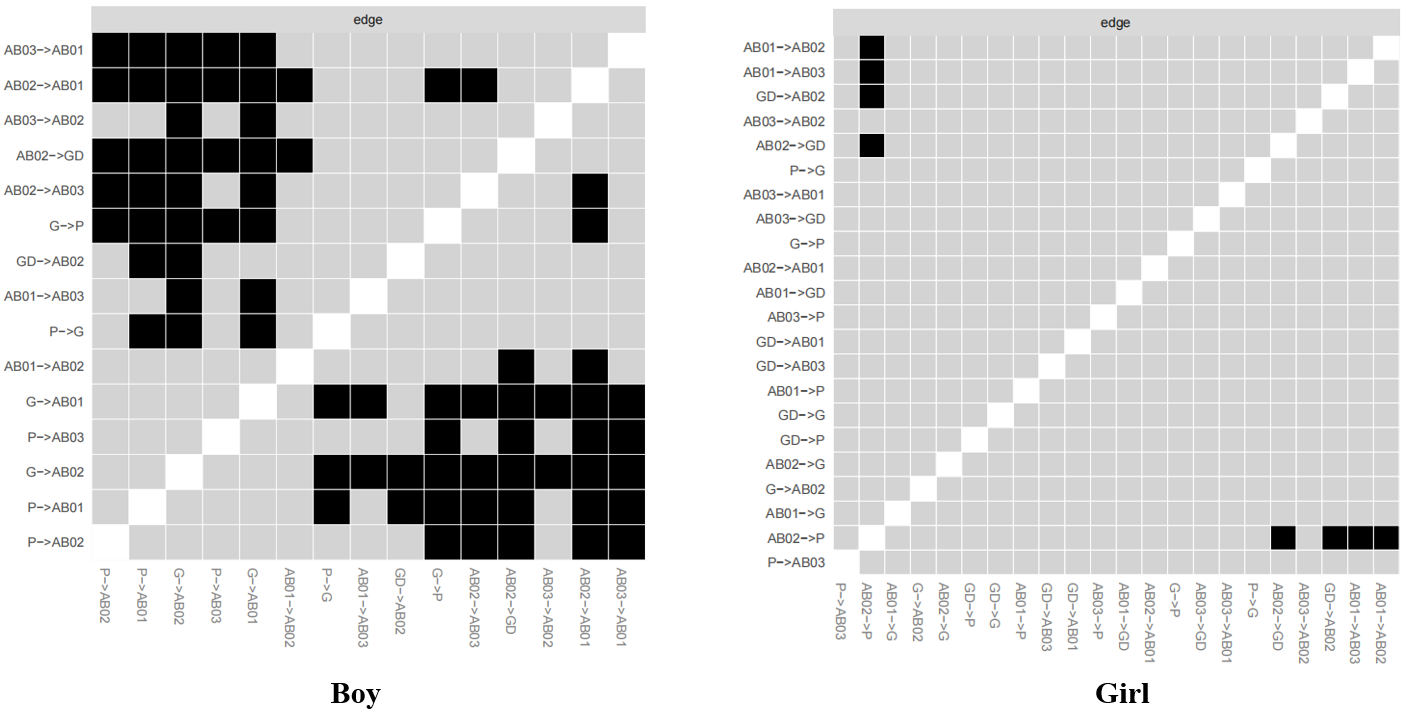


Fig. S2 The test for differences in the weights of the edges
